# Brigit Glorius, Izabela Grabowska-Lusińska & Aimee Kuvik (eds.): Mobility in Transition. Migration Patterns After EU Enlargement: Amsterdam University Press, Amsterdam

**DOI:** 10.1007/s10680-015-9352-2

**Published:** 2015-07-09

**Authors:** Paweł Strzelecki

**Affiliations:** Institute of Statistics and Demography, Warsaw School of Economics, Warsaw, Poland

The EU enlargement in the beginning of twenty-first century can be seen by demographers as a huge natural experiment regarding migrations. The book “Mobility in transition. Migration patterns after EU enlargement” describes some aspects of massive post-accession migration, but proposes also a reflection on how understanding of migration processes inside the EU has been changing based on the observations from Central and Eastern European countries (CEECs). Furthermore, the authors of the book suggest that observing mobility inside the EU only through the lens of long-term migration statistics and economic incentives is not sufficient and propose to refer to two concepts, “mobility in transition” and “liquid life”, in order to better understand not only post-accession migration but also post-crisis migration and other contemporary migrations of labour inside the European Union.

The term “mobility in transition”, which was also used in the title of the book, refers to so-called mobility transition hypothesis (Zelinsky 1971). According to this hypothesis, a personal mobility described as “widening range of options for locating and pattering one’s life” is one of the essential components of modernisation process. The new technologies indeed create opportunities to increase spatial mobility, but social acceptance of different forms of mobility seems to be also important.

The second term “liquid life” refers to post-modernistic sociological theory formulated by Bauman (2005). The theory claims that stable social institutions (class, family, local labour market, community, nation state) are of fading relevance and are being replaced by flexible institutions (reference group, relationships with “no strings attached”, global labour, individual, current country of residence). The transformation of traditional institutions that shaped migration in the past, advanced communication technologies and disappearance of internal borders have changed migration patterns in post-industrial societies. Contemporary migrants respond and adapt quickly to changing conditions in different labour markets and situations they operate.

Both sociological concepts seem to be the common language in interpreting the results by researchers from different countries that compiled the separate chapters of the “Mobility in transition…”. The book is structured in three parts. The chapters of the first part describe selected patterns of post-accession emigration. The descriptions itself largely repeat what we already know on this topic, but the value of these chapters lies in the way the authors interpret the findings by referring to the concept of the liquid migration. Furthermore, the authors of this part of the book point out problems with reliable classification of migratory moves using standard descriptions of migration (such as long and short term, settlement and seasonal migration) created by proliferation of new migration strategies. Liquid migration creates also challenges for modelling of the decision processes at the individual level. More and more migrants try to avoid long-term planning (intentional unpredictability), because the ability of quick, independent decisions can be treated as an asset in the rapidly changing contemporary world.

The second part of the book presents the insight into motives that explain why young, highly skilled people from CEECs relatively easily made decisions about emigration and accepted jobs significantly below their qualifications in destination countries. The advantage of this part of the book is that it does not oversimplify the problem by referring only to economic differences to explain the migration moves, but also points to labour market discrimination of migrants and glass ceiling in the receiving countries as well as to migration networks. It also sheds more light on the “brain waste” problem by showing that it is more complex and covers not only higher mobility of skilled young persons, but can also be seen through the lens of the contrast between the aims of immigration policies (filtering of the highly skilled) and different needs of the labour markets in receiving countries (higher demand for lower skilled jobs). On the other hand, some authors show that the problem of the “brain waste” seems to be rooted in the evolution of tertiary educational systems in CEECs that resulted in overprovision of graduates in e.g. marketing and management or pedagogics.

The third part of the book tries to answer the question about the reasons for return migration. This phenomenon of the fast but ephemeral change from large emigration to positive net migration was observed in many countries of origin of emigrants during the climax of the economic crisis. In the final chapter of the book the authors suggest that it can confirm the existence of liquid migration and “intentional unpredictability” of migrants. Indeed such a quick, huge adjustment at the time of the crisis seems to be characteristic for “liquid migrants” which are continuously ready to move. However, the problem is that besides the conclusions from qualitative analysis it is hard to propose a tool to measure the increasing share of persons that are “liquid migrants” and unfortunately such a tool was not proposed in this book either.

To sum up, “Mobility in transition…” should draw the attention of researchers who search for the ideas that explain contemporary migration behaviours in Europe. It can also be a valuable choice for specialists interested in a broader picture of the reasons for migration flows after the EU enlargement. This book offers many examples of qualitative studies in addition to basic but diversified quantitative analysis. However, those who want to find hard conclusions made on large data samples or applications of statistical modelling can be disappointed. Perhaps, this book can be just an inspiration for further advanced analysis.
